# A Case of Atopic Myelitis with Cervical Cavernous Angioma

**DOI:** 10.1155/2017/9506275

**Published:** 2017-07-03

**Authors:** Miyuki Fukuda, Hiroaki Manabe, Nobuhiro Sasaki, Masayuki Kuroda, Minoru Hoshimaru, Shigeo Ueda

**Affiliations:** Shin-Aikai Spine Center, Katano Hospital, Matsuzuka 39-1, Katano City, Osaka 576-0043, Japan

## Abstract

Atopic myelitis, a type of myelitis which appears in patients with elevated serum levels of immunoglobulin E (IgE), occurs more commonly in the cervical spinal cord, but this mechanism has not yet been elucidated. Herein, we experienced a case of atopic myelitis developed during the growth of cervical cavernous angioma caused by bleeding. A 37-year-old woman suffered from hand swelling caused by a house cat licking. At the same time when cavernous angioma had grown, she experienced a numbness in her four extremities, and multifocal peritumoral hyperintense spinal cord signals were seen. The diagnosis of atopic myelitis was made because we observed significantly elevated levels of specific IgE antibody to cat dander. Symptoms disappeared immediately after steroid pulse therapy. We subsequently resected a cavernous angioma, and eosinophil invasion was found inside it. This is the first case report of atopic myelitis which developed in association with spinal cord vascular lesions. A local blood-brain barrier breakdown due to hemorrhagic lesions of the spinal cord may have contributed to the onset of atopic myelitis.

## 1. Introduction

Atopic myelitis is a type of myelitis which has received attention along with the recent dramatic increase in atopic disease. It develops in patients with elevated serum levels of immunoglobulin E (IgE) or allergic predisposition, such as atopic dermatitis, allergic rhinitis, or bronchial asthma. Atopic myelitis occurs more commonly in the cervical spinal cord [1, 2]. There has been no report of atopic myelitis which occurs in association with vascular lesions of the brain and spinal cord. Herein, we report our experience with a case of atopic myelitis developed along with cervical cavernous angioma growth caused by bleeding.

## 2. Case Presentation

**Figure 1:**
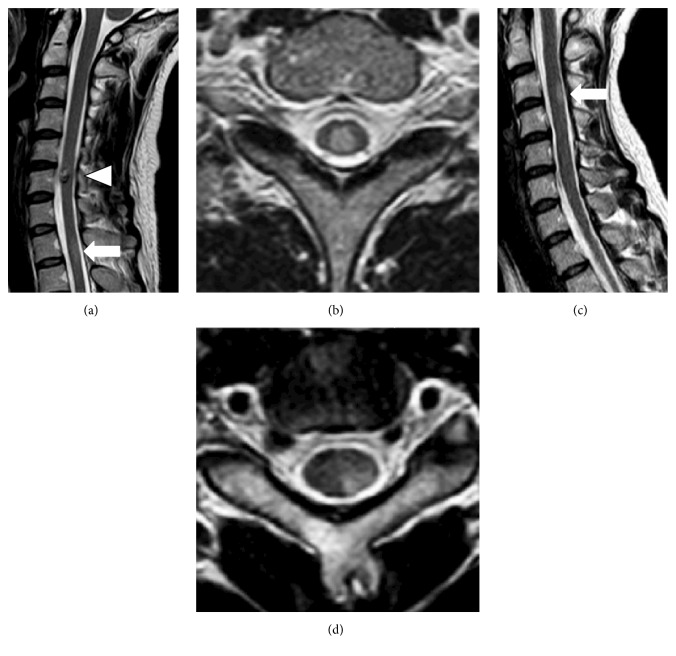
MRI images of atopic myelitis. (a) Cavernous angioma, which exhibits low intensity signal around the ventral side of the spinal cord at C6 vertebral level, is seen on T2WI (arrowhead). An intramedullary high-signal intensity area is found on the thoracic spinal cord at T1 vertebral level (arrow). (b) Axial image at T1 vertebral level on T2WI. An intramedullary change in signal intensity is observed on both sides of the spinal cord and around the dorsal side of the spinal cord. (c) A new intramedullary high-signal intensity area on T2WI is found on the dorsal side of the cervical spinal cord at C3 vertebral level (arrow). (d) Axial image at C3 vertebral level on T2WI. The lesion is locally observed on the left posterior funiculus.

At the age of 35, the patient developed cervical cavernous angioma on the right side of spinal cord on ventral aspect at the level of the right sixth cervical (C6) vertebra due to subarachnoid hemorrhage (SAH). A mild numbness of the four extremities remained after SAH. One year later, cavernous angioma had grown, and a numbness of the right upper extremity had been exacerbated. One year and a half after SAH, a numbness had extended from the trunk through both lower extremities, and a new T2-weighted intramedullary high-signal intensity area was found in spinal cord situated at the first to the second thoracic (T1-2) vertebral level (Figures 1(a) and 1(b)). A numbness of the left upper extremity appeared 1 year and 9 months after SAH, and a new T2-weighted intramedullary high-signal intensity area was also observed in the left side of spinal cord on dorsal aspect at the third to the fourth cervical (C3-4) vertebral level (Figures 1(c) and 1(d)).

## 3. A Previous History

The patient had no previous history of bronchial asthma, atopic dermatitis, and hay fever but experiences skin redness when a house cat licks her.

## 4. Neuroradiology

**Figure 2:**
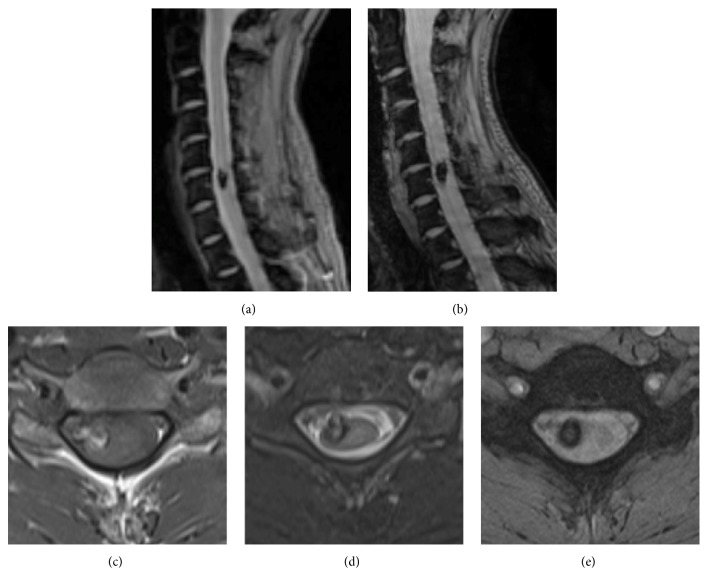
MRI images of cavernous angioma. (a) Cavernous angioma one year after SAH on T2-star sagittal image. (b) Preoperative T2-star sagittal image of cavernous angioma. The growth of the lesion is observed. (c), (d), (e) Preoperative axial image of cavernous angioma. Cavernous angioma extends laterally from the ventral side of the right half the spinal cord. Cavernous angioma is observed as a heterogenous lesion on T1 weighted image (c). Surrounding rim is seen on T2WI (d) and T2-star image (e).

Cavernous angioma was observed in the ventral side of the spinal cord at C6 vertebral level (Figure 1(a), white arrowhead). Cavernous angioma was present in the ventral side of the right half the spinal cord (Figures 2(c), 2(d), and 2(e)) and partially extramedullary (Figure 2(c)). Intramedullary high-signal intensity on T2 weighted image (T2WI) was observed in the spinal cord at T1-2 vertebral level (Figure 1(b)) at the same time that a numbness of both lower extremities appeared. A high-signal intensity area spread forward to the anterior part of the posterior funiculus of the spinal cord, but the anterior funiculus and lateral funiculus were retained. A new intramedullary high-signal intensity area on T2WI appeared at C3-4 vertebral level when the patient experienced a numbness of the left upper extremity (Figure 1(c)). A high-signal intensity area existed locally in the left posterior funiculus of the spinal cord (Figure 1(d)).

## 5. Blood Biochemical Findings

The followings were the laboratory results.

Complete blood count results were as follows: white blood cell count 4,400/*μ*L (neutrophils: 65.6%, lymphocytes: 27.3%, monocytes: 4.8%, eosinophils: 1.8%, and basophils: 0.5%), hemoglobin 14.9 g/dL (range: 11.5–15.4), hematocrit 43.7% (range: 33.0–44.0), platelet count 19.8 × 10^4^/*μ*L (range: 13.0 × 10^4^–37.0 × 10^4^), total protein 6.8 g/dL (range: 6.7–8.3), albumin 4.4 g/dL (range: 3.8–5.3), AST 11 IU/L (range: 8–38), ALT 8 IU/L (range: 4–44), CPK 51 IU/L (range: 43–165), BUN 12.5 mg/dL (range: 8.0–20.0), creatine 0.71 mg/dL (range: 0.4–0.8), Na 139 mEq/L (range: 135–147), K 4.2 mEq/L (range: 3.3–108), glucose 92 mg/dL (range: 70–110), and C-reactive protein 0.02 mg/dL (range: 0.0–0.3). A nonspecific IgE level was 497 IU/mL (normal range: 0–170), and a specific IgE level to cat dander was 85.5 UA/mL (reference range: 0–0.34). The patient exhibited patch test positivity to any of the following 12 substances:* Dermatophagoides pteronyssinus*,* Dermatophagoides farinae*, cat, dog,* Cynodon dactylon*,* Dactylis glomerata*,* Ambrosia artemisiifolia*,* Artemisia indica* var.* maximowiczii*,* Betula platyphylla*,* Cryptomeria japonica*,* Candida*, and* Alternaria*. Antibody against aquaporin-4 was negative. In the meantime, all following test results stayed within a normal range: serum complement level, complement components 3 and 4, rheumatoid factor, antinuclear antibody, anti-DNA antibody, anti-ribonucleoprotein antibody, anti-Smith antibody, anti-SSA and anti-SSB antibodies, anticardiolipin and anti–*β*2-glycoprotein I complex, and lupus anticoagulant.

## 6. Cerebrospinal Fluid (CSF) Analysis

Results of CSF analysis were as follows: specific gravity 1.006 (range: 1.005–1.007), protein 29 mg/dL (reference range: 8–43), glucose 68 mg/dL (reference range: 50–75), and total number of cells present 3/*μ*L (3 mononuclear cells, 0 polynuclear cells).

No oligoclonal IgG bands were detected.

## 7. Brain MRI

There were no abnormal findings including high-intensity signals in T2 weighted image.

## 8. Medical Therapy and Posttherapy Course

As a reason for multifocal hyperintense spinal cord signal, we considered the possibility of neuroinflammatory disorders, such as multiple sclerosis, neuromyelitis optica, or connective tissue disease, sarcoidosis, Behcet's disease, and so on [3–6], other than atopic myelitis, but we were skeptical about these possibilities according to the results of blood biochemical study. The diagnosis of atopic myelitis was made based on the blood biochemical findings, and steroid pulse therapy was performed using methylprednisolone (1000 mg/day, for 3 days). A numbness which has extended from the trunk through the left upper and lower extremities, along with the right lower extremity, disappeared immediately following the therapy. After hospital discharge, efforts were made to segregate a house cat and normalize skin barrier by increasing moisture retention.

## 9. Surgical Management and Postoperative Course

Atopic myelitis did not recur, but cavernous angioma was totally resected because its growth was identified (Figures 2(a) and 2(b)). Imaging findings presented cavernous angioma inside and outside the spinal cord. The patient had a good postoperative course and was discharged from the hospital with maintaining independence in the activities of daily living.

## 10. Histopathological Study

**Figure 3:**
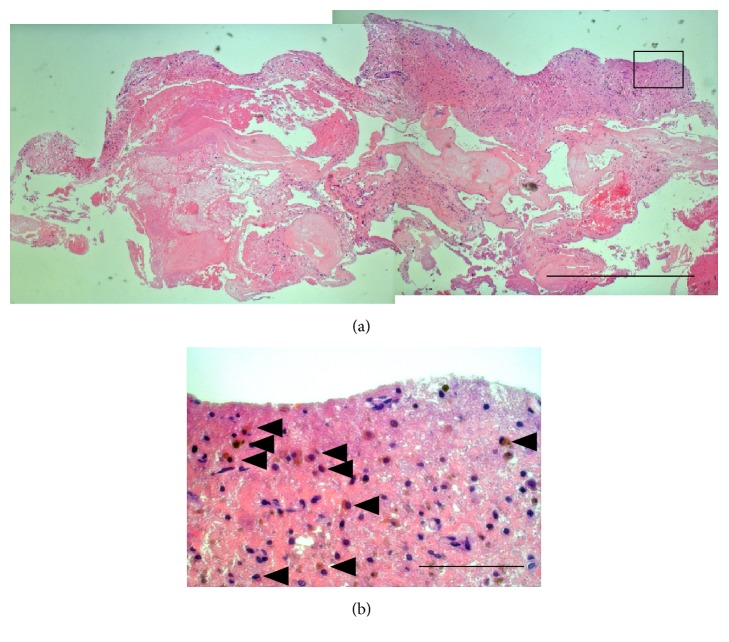
Pathological tissue images of the resected cavernous angioma (hematoxylin and eosin stain). (a) Various sizes of enlarged blood vessels are seen (bar = 100 *μ*m). The high cell density area (square in (a)) is shown enlarged in (b). (b) Cytoplasm in red, along with invasion by eosinophils which have an unevenly distributed nucleus, is observed (black arrowhead) (bar = 10 *μ*m).

Pathological examination of the resected cavernous angioma was performed. Hematoma was present inside the cavernous angioma, and irregularly enlarged vascular structure with thin vascular wall was observed (Figure 3(a)) as the typical pathological findings of cavernous angioma [7, 8]. Eosinophil invasion was seen in the high cell density area (Figure 3(b)).

## 11. Discussion

Atopic myelitis, an allergic disease with high serum levels of IgE and antigen-specific IgE, is the concept of disease advocated by Kira et al. in 1997 [9]. A nationwide survey on atopic myelitis has been conducted by Isobe et al. in Japan [2, 10].

Atopic myelitis is apt to develop after exacerbation of underlying allergic diseases which include atopic dermatitis, bronchial asthma, allergic rhinitis, food allergy, and allergic conjunctivitis [1, 10]. In some way, it seems that atopic myelitis results from the transmission of allergic reaction from the bloodstream to the spinal cord, but the detailed mechanism of atopic myelitis is still unknown.

Symptoms include paresthesia that is not associated with mobility impairment, and most patients initially present with a mild numbness. According to magnetic resonance imaging (MRI) study, high-intensity signal on T2WI is present around the posterior funiculus of the cervical spinal cord [1], gadolinium enhancement and edematous changes in the spinal cord are found in the acute stage, and blood-brain barrier (BBB) breakdown is locally observed [11]. Atopic myelitis is pathologically considered as vasculitis in the presence of invasion by eosinophils which seem to act as effector cells in myelitis [11–13]. Steroid pulse therapy is used to treat atopic myelitis, but the efficacy rate is about 70%. Plasma exchange is used if steroid pulse therapy is ineffective [2, 14].

Cavernous angioma is a type of vascular malformation which grows while repeating extremely light bleeding. Pathologically speaking, vascular structure with irregularly enlarged, thin-walled vessels was observed [7, 8]. Cavernous angioma is sometimes identified as the cause of SAH, and extremely light bleeding can be confirmed by T2-star-weighted MRI [15]. Because cavernous angioma grows while bleeding, the resection of cavernous angioma is considered when neurological deficits are observed after the growth [16, 17].

In this case, the patient did not present with previous major atopic disease or exacerbation of atopic disease, such as atopic dermatitis or bronchial asthma, but because of high serum levels of IgE along with a detailed history of the patient, we diagnosed cat allergy, of which the patient herself was not aware. With regard to a miner allergy, attention is required because a detailed history needs to be taken from the patient. In addition, because cavernous angioma had caused SAH and afterward had grown while repeating mild bleeding, BBB breakdown might have occurred in the cervical spinal cord. During the growth of cavernous angioma, the patient developed atopic myelitis in multiple areas adjacent to the angioma, and eosinophil invasion was observed inside the resected cavernous angioma; therefore, BBB breakdown resulting from vascular lesions associated with bleeding may have facilitated the transmission of allergic reaction from the bloodstream to the spinal cord.

## 12. Conclusion

This article is the first case report of atopic myelitis which developed in association with vascular lesions of the central nervous system. BBB breakdown due to angioma may have been involved in the transmission of allergic reaction to the spinal cord.

## References

[1] Kira J.-I. (2002). Atopy and neural damage. *Internal Medicine*.

[2] Isobe N., Kira J., Kawamura N., Ishizu T., Arimura K., Kawano Y. (2009). Neural damage associated with atopic diathesis: a nationwide survey in Japan.. *Neurology*.

[3] Isobe N., Kanamori Y., Yonekawa T. (2012). First diagnostic criteria for atopic myelitis with special reference to discrimination from myelitis-onset multiple sclerosis. *Journal of the Neurological Sciences*.

[4] Hebel R., Dubaniewicz-Wybieralska M., Dubaniewicz A. (2015). Overview of neurosarcoidosis: recent advances. *Journal of Neurology*.

[5] Sastre-Garriga J., Tintorè M., Montalban X. (2016). MRI criteria for the diagnosis of multiple sclerosis: MAGNIMS consensus guidelines. *The Lancet Neurology*.

[6] Al-Araji A., Kidd D. P. (2009). Neuro-Behçet's disease: epidemiology, clinical characteristics, and management. *The Lancet Neurology*.

[7] Abe M., Tabuchi K., Tanaka S. (2004). Capillary hemangioma of the central nervous system. *Journal of Neurosurgery*.

[8] Wu L., Deng X., Yang C., Xu Y. (2013). Intramedullary spinal capillary hemangiomas: clinical features and surgical outcomes. *Journal of Neurosurgery: Spine*.

[9] Kira J.-I., Yamasaki K., Kawano Y., Kobayashi T. (1997). Acute myelitis associated with hyperIgEemia and atopic dermatitis. *Journal of the Neurological Sciences*.

[10] Osoegawa M., Ochi H., Minohara M. (2003). Myelitis with atopic diathesis: a nationwide survey of 79 cases in Japan. *Journal of the Neurological Sciences*.

[11] Kikuchi H., Osoegawa M., Ochi H. (2001). Spinal cord lesions of myelitis with hyperIgEemia and mite antigen specific IgE (atopic myelitis) manifest eosinophilic inflammation. *Journal of the Neurological Sciences*.

[12] Osoegawa M., Ochi H., Kikuchi H. (2003). Eosinophilic myelitis associated with atopic diathesis: a combined neuroimaging and histopathological study. *Acta Neuropathologica*.

[13] Park C. W., Choe W. J., Chun Y. I. (2012). Eosinophilic myelitis in the cervical cord mimicking intramedullary cord tumor. *Journal of Korean Neurosurgical Society*.

[14] Murai H., Arahata H., Osoegawa M. (2004). Effect of immunotherapy in myelitis with atopic diathesis. *Journal of the Neurological Sciences*.

[15] Hegde A. N., Mohan S., Lim C. C. T. (2012). CNS cavernous haemangioma: 'popcorn' in the brain and spinal cord. *Clinical Radiology*.

[16] Gross B. A., Du R., Popp A. J., Day A. L. (2010). Intramedullary spinal cord cavernous malformations.. *Neurosurgical focus*.

[17] Badhiwala J. H., Farrokhyar F., Alhazzani W. (2014). Surgical outcomes and natural history of intramedullary spinal cord cavernous malformations: a single-center series and meta-analysis of individual patient data. *Journal of Neurosurgery: Spine*.

[18] Minoru H., Nobuhiro S., Masayuki K., Shigeo U. A case of atopic myelitis with cervical cavernous angioma.

